# The Associations of HLA-A*02:01 and DRB1*11:01 with Hepatitis C Virus Spontaneous Clearance Are Independent of *IL28B* in the Chinese Population

**DOI:** 10.1038/srep31485

**Published:** 2016-08-11

**Authors:** Jieting Huang, Ke Huang, Ru Xu, Min Wang, Qiao Liao, Huaping Xiong, Chengyao Li, Xi Tang, Zhengang Shan, Ming Zhang, Xia Rong, Kenrad Nelson, Yongshui Fu

**Affiliations:** 1 Guangzhou Blood Center, Guangzhou, Guangdong, China; 2 The Key Medical Disciplines and Specialties Program of Guangzhou, Guangdong, China; 3 Department of Transfusion Medicine, School of Biotechnology, Southern Medical University, Guangzhou, Guangdong, China; 4 Department of Epidemiology and Biostatistics, Faculty of Infectious Diseases, University of Georgia, Athens, GA, USA; 5 Department of Epidemiology, Bloomberg School of Public Health, Johns Hopkins University, Baltimore, Maryland, USA

## Abstract

Spontaneous clearance of hepatitis C virus (HCV) occurs in 10–40% of the infections. Specific human leukocyte antigen (HLA) alleles have been identified in associating with HCV clearance. However, data on the association of HLA with the spontaneous clearance of HCV are scarce in the Chinese population. In the current study we studied the HLA class I and class II genes in 231 Chinese voluntary blood donors who had cleared HCV infection spontaneously compared to 429 subjects with chronic HCV infections. We also studied their *IL28B* SNP (rs8099917) genotype, since a number of investigators have found a strong association of *IL28B* with spontaneous or treatment induced HCV clearance. We found that HLA-A*02:01 and DQB1*05:02 distributed differently between the two groups after Bonferroni correction (odds ratio [OR] = 1.839, *Pc* = 0.024 and OR = 0.547, *Pc* = 0.016, respectively). Multivariate logistic regression analysis suggested that A*02:01 and DRB1*11:01 (OR = 1.798, *P* = 0.008 and OR = 1.921, *P* = 0.005, respectively) were associated with HCV spontaneous clearance, independent of age, gender and *IL28B* polymorphism. We concluded that in the Chinese population, HLA-A*02:01 and DRB1*11:01 might be associated with the host capacity to clear HCV independent of *IL28B*, which suggesting that the innate and adaptive immune responses both play an important role in the control of HCV.

Globally, hepatitis C virus (HCV) infection contributes significantly to chronic liver diseases, including liver fibrosis, cirrhosis, hepatocellular carcinoma or liver failure^1^. An estimated of 170–210 million people worldwide are HCV seropositive^2^, and the HCV incidence in China was over 200,000 infections per year from 2012 to 2015^3^.

The spontaneous clearance of HCV occurs in 10–40% of the infections worldwide^4^. The mechanisms of HCV clearance are not well elucidated. Clearance of HCV has been associated with several demographic and host factors, including female gender, younger age, Caucasian or Hispanic ethnicity, co-infection with hepatitis B virus (HBV) and host immunity^5^,^6^,^7^. Evidence that host genetic constitution was associated with HCV clearance was obtained from studies of single nucleotide polymorphisms (SNPs) linked to the interferon lambda 3 (*IFNL3*) gene^8^,^9^. The *IFNL3* gene, also known as interleukin-28B (*IL28B*), encodes an innate interferon IFNλ3, which has antiviral activity upon the activation of the JAK-STAT pathway that up-regulates the expression of interferon-stimulated genes (ISGs)^10^. Although the underlying mechanism for this effect is not well understood, the association of *IL28B* SNPs with HCV spontaneous and treatment induced clearance is clearly defined^8^,^9^,^11^, which has prompted the usage of *IL28B* SNPs in designing the HCV therapy strategy and to predict treatment outcome^12^.

The *IL28B* polymorphism has also been used to predict the probability of HCV spontaneous clearance^11^,^13^. However, the combination of *IL28B* SNPs with HLA-DQB1*03:01 predicted only about 15% of the HCV spontaneous clearance cases in subjects of European and African ancestry^11^. In addition, a racial difference was associated with the frequency of HCV spontaneous clearance and that was unexplained by the difference of *IL28B* polymorphisms in diverse populations. For example, Caucasians and Hispanics were predicted to have 3–4 times higher HCV clearance proportion than those of the African ancestry^6^,^14^,^15^, while the frequency of *IL28B* rs12979860-C allele, which was in favor of HCV clearance, was 55–65% in the European and American subjects, compared with 30–40% in the African population^8^. Additionally, the frequency of rs12979860-C allele was over 90% in the Chinese population^8^,^16^, however only 20% of HCV infected subjects spontaneously cleared their HCV infection^17^, which was lower than that of Caucasians. HCV clearance can be partially explained by the distribution of *IL28B* SNPs, however other factors are involved.

Cellular immune responses mediated by activated CD4+ and CD8+ T cells are critical for HCV clearance^18^,^19^. Strong and broad CD4+ helper and CD8+ effector T cell responses are associated with HCV clearance^20^,^21^; progression to chronicity has been associated with the functional exhaustion of HCV-specific CD4+ and CD8+ T cells^22^. The activation of T cells is determined by the recognition of T cell receptors and the human leukocyte antigen (HLA) presented on the surface of the infected hepatocytes^20^. Previous studies have identified specific HLA class I alleles associated with HCV clearance, such as HLA-A*03, B*27, B*57, Cw*01 and Cw*04^13^,^23^,^24^,^25^. HLA class II genes, including DRB1*11:01 and DQB1*03:01, have been associated with HCV clearance, while DRB1*03:01 and DQB1*02:01 have been associated with persistent HCV infection^24^,^26^. It should be noted that the association of HLA and HCV spontaneous clearance seems to be race specific^26^,^27^. Thio *et al*. identified HLA-DQB1*03:01 to associate with HCV clearance in black but not white subjects, while the association of DRB1*01:01 and DRB1*03:01 was associated with HCV clearance in white but not black subjects^26^. Wang *et al*. reported opposite associations of HLA-A*02 and DRB1*12 with resolution of HCV infection between Caucasians and non-Caucasians^27^. However, there is little information on the association of specific HLA alleles with the spontaneous clearance of HCV in the Chinese population, despite the fact that the HCV prevalence is high in China. In addition, only a few studies have studied the combined association of the *IL28B* genotypes and HLA alleles with HCV clearance^13^.

In this study, we aimed to identify specific HLA class I (A, B and C) and class II alleles (DPB1, DQB1 and DRB1) associated with HCV spontaneous clearance among the 231 blood donors with HCV spontaneous clearance and 429 donors with persistent infections. In comparison, part of the genotyping information for HLA-A, B and DRB1 genes in the persistent infection donors were included from our previous report^28^. As far as we are aware, this study is the first to report the combined association of *IL28B* and HLA alleles with the spontaneous clearance of HCV in a large Chinese population.

## Results

### Epidemiological characteristics of the study subjects

A total of 1493 HCV seropositive voluntary blood donors were identified and recruited during July 2009 through March 2015. Among them, 231 donors who were anti-HCV positive but HCV RNA negative represented the spontaneous clearance group, while 1262 subjects with both anti-HCV and HCV RNA positive represented the persistent infection group. The overall spontaneous clearance rate was 15.5%, and male donors showed significant lower clearance rate than female donors (12.7% vs. 26.8%, *P* = 2.03E-9 in Chi-square test, Table S1). In order to assess the association of HLA alleles and HCV clearance, all the subjects with HCV clearance (n = 231) were enrolled, while 429 out of 1262 subjects represented the persistent infection group were sampled using systematic random sampling method. As shown in Table 1, all the study subjects were Chinese and most were of Han ethnicity. 34.2% (79/231) of the donors who spontaneously cleared the virus were female, compared with 17.2% (74/429) in those with persistent infection. The male/female ratio was significantly lower in the spontaneous clearance group (*P* = 8.58E-7 in Chi-square test). Additionally, the subjects in the clearance group were significantly younger than those in the persistent infection group (*P* = 3.29E-4 in T-test).

### Association of HLA class I and class II alleles with HCV spontaneous clearance

First, we determined the frequency of HLA class I and class II alleles in both HCV spontaneous clearance and persistent infection groups. Reliable genotyping for HLA-A, B and DRB1 loci was obtained for all 660 blood donors, while three cases, six cases and one case could not be typed for the C, DPB1 and DQB1 loci, respectively (Table S2). The HLA genotyping identified 13, 21, 13, 12, 13 and 19 specific alleles (frequency >1%) for HLA-A, B, C, DPB1, DQB1 and DRB1 loci, respectively (Figures S1–S6). When assessing the association of HLA with HCV clearance, we focused on the alleles with >5% frequency, because low-frequency alleles were more prone to spurious associations in an exploratory analysis. One allele in HLA class I loci (Table 2) and five in class II loci (Table 3) were found to be distributed differently between the spontaneous clearance group and the persistent infection group. HLA-A*02:01 (odds ratio [OR] = 1.839, 95% confidence interval [CI] = 1.212–2.791, *P* = 0.004), DQB1*03:01 (OR = 1.398, 95% CI = 1.060–1.843, *P* = 0.017) and DRB1*11:01 (OR = 1.820, 95% CI = 1.172–2.826, *P* = 0.007) were more prevalent in donors who cleared HCV. In contrast, DQB1*05:02 (OR = 0.547, 95% CI = 0.370–0.809, *P* = 0.002), DRB1*04:05 (OR = 0.514, 95% CI = 0.288–0.916, *P* = 0.022) and DRB1*15:01 (OR = 0.657, 95% CI = 0.448–0.964, *P* = 0.031) were more frequently found in subjects who had developed a persistent infection. After the Bonferroni correction for multiple comparisons, HLA-A*02:01 (*Pc* = 0.024) and DQB1*05:02 (*Pc* = 0.016) were found to be significantly associated with the spontaneous clearance of HCV. It should be noted that although the association of HLA-B*57 and HCV clearance has been widely reported^23^,^25^,^29^,^30^, the frequency of B*57 was very low (0.4% and 0.6% in the clearance and persistence group, respectively, data not shown) and the association of B*57 and HCV clearance was not significant in our study subjects (*P* = 0.999, data not shown).

### HLA alleles associate with HCV spontaneous clearance when the effect of *IL28B* SNP is considered

The association of *IL28B* SNPs with spontaneous and treatment induced HCV clearance has been extensively studied^8^,^9^,^11^. In order to address whether the associations of HLA alleles with HCV clearance were *IL28B*-independent, we genotyped two SNPs related to the *IL28B* gene, rs8099917 and rs12979860. The frequency of rs8099917-T allele, which favored HCV clearance, was greater than 90% in both groups, and it was significantly higher in the HCV clearance group compared to the persistent infection group (Table 4, 97.6% vs. 91.3%, *P* = 7.97E-6 in Chi-square test). The majority of the donors carried the favorable TT genotype, which was more frequent in the spontaneous clearance group than in the persistent infection group (95.7% vs. 82.5%, *P* = 1.50E-6 in Chi-square test). Similar results were obtained for rs12979860 (Table S3, *P* = 1.04E-5 and *P* = 2.03E-6 in Chi-square test for allele and genotype, respectively). Rs8099917 was in strong linkage disequilibrium with rs12979860 (Spearman’s rho = 0.993, data not shown).

To address the impact of *IL28B* on the association of HLA and HCV clearance, we stratified the study subjects according to their rs8099917 genotype and re-analyzed the six specific HLA alleles described above, i.e., A*02:01, DQB1*03:01, DQB1*05:02, DRB1*04:05, DRB1*11:01 and DRB1*15:01. As shown in Table S4, among donors who carried the protective *IL28B* TT genotype, HLA-A*02:01, DQB1*03:01 and DRB1*11:01 (OR = 1.673, *P* = 0.016; OR = 1.555, *P* = 0.002 and OR = 1.851, *P* = 0.007, respectively) were more frequent in the spontaneous clearance group, while DQB1*05:02 and DRB1*04:05 (OR = 0.582, *P* = 0.008 and OR = 0.516, *P* = 0.023, respectively) were more prevalent in the persistent infection group. DRB1*15:01 appeared to be less frequent in the spontaneous clearance group than in the persistence group (9.3% vs. 12.6%), but the difference was not statistically significant (*P* = 0.086). No significant association was found in the *IL28B* deleterious genotype (TG + GG), which was probably due to the small sample size in the clearance group (n = 10). These results suggested an *IL28B*-independent association between HLA class I and class II alleles and HCV spontaneous clearance.

### The association of HLA-A*02:01 and DRB1*11:01 with HCV spontaneous clearance were independent of gender, age and *IL28B*

It has been established that subjects who are females and younger are more likely to clear HCV spontaneously^6^,^15^,^31^. In this study, the male/female ratio and age were also significantly different between the spontaneous clearance group and the persistent infection group (Table 1). We then investigated whether the associations of these HLA alleles and HCV spontaneous clearance were independent of age, gender and *IL28B* SNP. A multivariate logistic regression analysis incorporating potential confounding factors including age, gender and *IL28B* was done and the results were shown in Table 5. The following variables were found independently associated with HCV clearance: gender (OR = 0.405, 95% CI = 0.310–0.531, *P* = 5.05E-11), age (OR = 1.035, 95% CI = 1.021–1.048, *P* = 3.02E-7), rs8099917 (OR = 3.842, 95% CI = 1.993–7.405, *P* = 5.82E-5), HLA-A*02:01 (OR = 1.798, 95% CI = 1.169–2.766, *P* = 0.008) and DRB1*11:01 (OR = 1.921, 95% CI = 1.223–3.017, *P* = 0.005). In contrast, DQB1*03:01 (*P* = 0.244), DQB1*05:02 (*P* = 0.051), DRB1*04:05 (*P* = 0.069) and DRB1*15:01 (*P* = 0.305), although distributed differently in the two groups, were not independently associated with HCV clearance. Taken together, our results suggested that HLA-A*02:01 and DRB1*11:01 associated with HCV clearance independent of age, gender and *IL28B*.

### Prediction of HCV spontaneous clearance by HLA and *IL28B*

Since there is a high prevalence of an *IL28B* favorable allele/genotype (Table 4) but a relatively low spontaneous HCV clearance level in the Chinese population^17^, the associations of *IL28B* and other host factors with viral clearance should be evaluated. We evaluated the sensitivity and positive predictive value (PPV) of *IL28B* and the two HLA alleles described above (HLA-A*02:01 and DRB1*11:01) with HCV clearance. As shown in Table S5, after adjusting for age and gender, the sensitivity of *IL28B* on HCV clearance was 22.9%, in comparison with 26.0% for the HLA alleles. The PPV was comparable for *IL28B* and HLA alleles (57.0% and 55.8%, respectively). When considering *IL28B* SNP and HLA-A*02:01 and DRB1*11:01 together, a slightly additive effect was observed (sensitivity = 28.1%, and PPV = 57.8%). In summary, considering both the *IL28B* genotype and HLA genes together can improve the prediction of the probability of HCV spontaneous clearance.

## Discussion

In a previous study we reported the association of HLA with HCV infection^28^. In this study, the parts of HLA-A, B and DRB1 genotypes obtained previously from blood donors^28^ were used as persistent HCV infection group in comparison with spontaneous HCV clearance. We aimed to examine the association of HLA class I and class II alleles with HCV spontaneous clearance in the Chinese population. In particular, we aimed to evaluate the association of HLA alleles in the context of *IL28B* polymorphisms, which has been shown to be strongly correlated with HCV clearance. To our knowledge, the present study is the first of its kind among the Chinese population. The study subjects had experienced an asymptomatic HCV infection at the time of enrollment and were treatment naïve, thus making it ideal for studying of the HCV natural history.

In our initial analysis, higher percentage of female presented in the HCV spontaneous clearance group compared with the persistent infection group (34.2% vs.17.2%, Table 1), indicating that female gender were more likely to clear HCV than male. Actually, the spontaneous clearance rate of HCV for female was higher than male (26.8% vs. 12.7%, Table S1). Our results demonstrated that gender influenced on HCV clearance, which was in line with that previously described in other studies^5^,^6^,^7^. Besides, subjects in the clearance group tended to be younger than those in the persistence group (Table 1), which was also in accordance with previous reports^5^,^6^,^7^. Therefore, these two host factors must be considered when assessing the association of HLA and HCV clearance.

We applied high resolution HLA genotyping to assess and compare their frequencies between the spontaneous clearance group and the persistent infection group. Six alleles (A*02:01, DQB1*03:01, DQB1*05:02, DRB1*04:05, DRB1*11:01 and DRB1*15:01) were distributed differently between the two groups, but only A*02:01 and DQB1*05:02 reached statistical significance after Bonferroni correction (*Pc *< 0.05), which was calculated by dividing the *P* value by the number of alleles with frequency >5%. However, this association considered only the frequency of the HLA alleles. The confounding effects of other potential factors remained unknown. It has been well established that the *IL28B* polymorphism is strongly associated with the HCV natural history and treatment outcome^5^,^8^,^9^,^11^. However, only a few studies have considered the effect of *IL28B* in assessing the association of HLA and HCV clearance^13^. In addition, as previously described, confounding factors including gender and age should be included to determine the association of HLA and HCV clearance. Therefore, multivariate logistic regression was performed using the age, gender, *IL28B* and HLA alleles with *P *< 0.05 by univariate analysis without correction (A*02:01, DQB1*03:01, DQB1*05:02, DRB1*04:05, DRB1*11:01, DRB1*15:01) as candidates. Multivariate logistic regression analysis supported the association of A*02:01 and DRB1*11:01 with HCV clearance, independent of *IL28B*, gender or age.

A number of studies have shown associations of HLA class I and class II alleles with HCV natural history or treatment outcome. We compared our findings with that of other studies (Table 6). HLA-A*02 is one of the most frequent HLA alleles in the population of European/Caucasian descent as well as in Chinese and Japanese^32^,^33^. This allele appeared to be associated with protection against chronic HCV since it was more prevalent among the healthy than the HCV-infected population^34^. HLA-A*02 may also be an independent predictor for an effective immune responses to interferon and ribavirin therapy in Chinese subjects who were infected with chronic hepatitis C^33^. Interestingly, A*02 seemed to be associated with lower clearance of HCV among Caucasians and higher clearance of HCV among non-Caucasians^27^. Here we reported the first study in which A*02:01 is associated with HCV spontaneous clearance in the Chinese population, and proposed the inclusion of this allele in assessing the risk of developing chronic hepatitis C in subjects of different ethnical origins. It should be noted that the current study applied high resolution 4-digit genotyping, which provided more accurate and robust data compared to the traditional 2-digit level that was commonly used in previous reports^23^,^24^,^25^,^33^. Our high resolution HLA genotyping results showed that A*02:01, rather than A*02:03 or A*02:07, was significantly associated with HCV clearance (Table 2). Such specific association would be missed if only 2-digit HLA genotyping was used.

Several studies had demonstrated that HLA-B*57 is associated with HCV spontaneous clearance^23^,^25^,^29^,^30^,^35^. The carriage of B*57 and the recognition of B*57-restricted T-cell responses have been associated with HCV clearance^29^. However, this association was not found in this study, probably because of the very low prevalence of the B*57 allele in the Chinese population. It was found in only 0.4% and 0.6% of the two groups in this study, whereas it was present in 10–15% of the allele in American or African populations^23^,^25^.

The protective role of HLA-DRB1*11^7^,^34^,^36^,^37^,^38^,^39^,^40^ and DQB1*03^7^,^26^,^27^,^34^,^36^,^37^,^38^,^41^,^42^,^43^ with HCV clearance has been consistently reported across diverse populations. Our results supported the association of DRB1*11:01 with HCV spontaneous clearance (Table 5). However, although DQB1*03:01 seemed more prevalent in subjects who cleared HCV than those with persistent infection, the association was marginal (Table 3) and not independent of *IL28B*, gender and age (Table 5). The discrepant results on DQB1*03:01 between published data and this study can not be simply explained by the difference of the studied population. Actually, we observed that DQB1*03:01 associated with *IL28B*, A*02:01 and DRB1*11:01 (r = 0.08, *P* = 0.004; r = 0.093, *P* = 0.001 and r = 0.304, *P* = 1.38E-29, respectively, data not shown). In our stratified analysis, the association of DQB1*03:01 and HCV clearance was stronger in subjects carried *IL28B* TT genotype (Table S3) or A*:02:01 (data not shown), indicating that DQB1*03:01 was associated with HCV clearance when confounding factors were considered. In addition. DRB1*11:01 and DQB1*03:01 are well known to be highly associated because of linkage disequilibrium^44^ and this association was verified in our data (D’ = 0.983, *P* = 1E-37, data not shown). Therefore, our logistic regression model incorporating DRB1*11:01 excluded DQB1*03:01 as associated with HCV clearance. Taken together, our results verified the association of DRB1*11:01 on HCV spontaneous clearance in the Chinese population, and we speculated that the association of DQB1*03:01 with HCV clearance may be masked by *IL28B*, HLA-A*02:01 and DRB1*11:01.

Our results also suggested that subjects carrying HLA-DQB1*05:02 were at greater risk of developing chronic hepatitis C (Table 3). This allele was marginally associated with failure of HCV resolution (*P* = 0.051, Table 5). This contrasted with the study of Congia *et al*., which reported an association between DQB1*05:02 and HCV clearance in the presence of DRB1*16:01^45^. It should be pointed out that Congia’s study subjects were of different ethnic origin compared to ours, and the sample size in our study was larger than Congia’s study. In addition, HLA-DQB1*05^27^ and DQB1*05:01^26^ were also reported to be associated with the spontaneous clearance of HCV, which disagreed with our results. We suspect that the slight difference on the studied alleles (DQB1*05, 05:01 and 05:02) and the subjects of different ethnicity may account for the discordant results. Further investigation is required to determine the role of DQB1*05, 05:01 or 05:02 on HCV outcome in diverse ethnical groups.

The association of DRB1*15 with HCV spontaneous clearance is unclear. While this allele was not associated with HCV clearance in this study, Lechmann *et al*. reported an increased frequency of DRB1*15 in German HCV patients with resolution of HCV infection^46^; this was supported by McKiernan’s study of a group of Irish women who infected HCV from a single source^24^. However, the study conducted by Ksiaa *et al*. in Tunisia found that DRB1*15 occurred more often in chronic HCV infected subjects compared with those with viral clearance^47^. The role of DRB1*15 in HCV outcome is still elusive and seems to be population specific, which requires further study to determine.

Despite a strong correlation of *IL28B* SNP with spontaneous and treatment induced HCV clearance, *IL28B* SNP can only predict about 15% of the spontaneous clearance^11^ and two-thirds of the sustained virological responses to antiviral therapy among HCV infected patients^10^. In the Chinese population, the favorable *IL28B* allele/genotype did not appear to be a good predictor for HCV spontaneous clearance, because its frequency was over 90% in both subjects who had cleared HCV and those with persistent infections (Table 4 and ref. 16). The association of rs8099917 and HCV clearance was more apparent among the minority of subjects with the unfavorable *IL28B* genotype. Subjects with this *IL28B* genotype were 3.6-fold more likely to have persistent infection than spontaneous clearance (i.e. 8.7% vs. 2.4%, Table 4). It is plausible that factors other than *IL28B* SNP are involved in HCV clearance in the Chinese population. To explore this possibility, we assessed the prediction of HCV spontaneous clearance by optimizing the multivariate model with rs8099917 and HLA alleles (A*02:01 and DRB1*11:01). Both *IL28B* and HLA alleles had comparable predictive values, while a slightly additive effect was observed when both *IL28B* and HLAs were considered (Table S4). Our results are similar to those reported by Fitzmaurice *et al*. in Irish women^13^. Taken together, the independent associations of *IL28B* SNP and HLA with HCV clearance supported the importance of the integration of host innate and adaptive immune genes on the control of HCV viremia.

In conclusion, our results found that in the Chinese population, HLA-A*02:01 and DRB1*11:01 were individually associated with HCV spontaneous clearance, even after the effect of *IL28B* was considered. These two HLA alleles, together with *IL28B*, should be considered in the prediction of the spontaneous HCV clearance.

## Methods

### Study subjects

All the study subjects were voluntary blood donors who passed pre-donation questionnaire and donated their blood at Guangzhou Blood Center between July 2009 and March 2015. Before their blood donation, all individuals were requested to complete a blood donation health consent form for the study participation. Blood samples collected at donation were used to test HCV and other pathogens (see “detection of HCV infection” section below). A follow-up examination for the HCV seropositive donors was performed 6 months after their blood donation. Physicians interviewed the participants to assure their understanding of the informed consent for both enrollment and follow-up examination. Donors who were anti-HCV and HCV RNA positive at both enrollment and follow-up examination represented the HCV persistent infected group, while those who with undetectable HCV RNA but positive for anti-HCV at both tests represented the HCV spontaneous clearance group. All the subjects with HCV clearance (n = 231) were enrolled, while 429 subjects of the persistent infection group were sampled using systematic random sampling method from 1262 persistent infected blood donors who were recruited during the same period. This study strictly followed the ethical guidelines of the 1975 Declaration of Helsinki and was approved by the Medical Ethics Committee of Guangzhou Blood Center.

### Detection of HCV infection

Screening of HBV, HCV, human immunodeficiency virus (HIV) and Treponema pallidum (TP) and follow-up examination was done as previously described^28^. Briefly, two independent enzyme-linked immunosorbent (EIA) kits were applied to detect anti-HCV (Kehua HCV EIA assay, Kehua Biotech Co. Ltd., Shanghai, China and Abbott HCV EIA 2.0, Abbott Laboratories, Chicago, IL, USA). HBV, HIV and TP were also detected by two EIA assays. HBV DNA, HCV RNA and HIV RNA were detected using nucleic acid testing (NAT, Procleix Ultrio Assay; Gen-Probe, San Diego, CA, USA) according to the manufacturers’ instruction. All the enrolled blood donors were positive for anti-HCV and negative for anti-HIV, HBV surface antigen (HBsAg), anti-TP antibody, as well as HBV and HIV nucleic acids. EIA-reactive and HCV RNA negative specimens were subsequently tested by a third-generation recombinant immunoblot assays (RIBA. HCV Blot 3.0, MP Diagnostics, Singapore) to determine the presence of anti-HCV antibody. Donors with HCV RNA negative and RIBA positive in donation and follow up examination were assigned to the spontaneous clearance group, while those who had detectable HCV RNA in the two examinations were assigned to the persistent infection group.

### *IL28B* genotyping

Two *IL28B* SNPs, rs8099917 and rs12979860, were genotyped by PCR and Sanger sequencing. First, genomic DNAs were extracted from whole blood samples using QuickGene-610L system (Fujifilm, Tokyo, Japan) following manufacturers’ instruction. The DNA fragments containing rs8099917 or rs12979860 were amplified using GoTaq Colorless Master Mix (Promega, Madison, WI, USA) with the presence of 0.5 μM primers. The primer sequences were designed as following: (5′-3′): rs8099917-F, CCACTTCTGGAACAAATCGTCCC; rs8099917-R, TCAACCCCACCTCAAATTATCCT; rs12979860-F, GGACGAGAGGGCGTTAGAG; rs12979860-R, GGCTCAGGGTCAATCACAG. The length of amplicons containing rs8099917 and rs12979860 were 294 and 309 bp, respectively. The amplicons were subsequently applied to Sanger sequencing in both directions by ABI 3730 DNA Sequencer (Applied Biosystems, Foster City, CA, USA). The SNP genotype was determined by assembling the obtained sequences using SeqMan software (DNAStar, Inc. Madison, WI, USA).

### HLA genotyping

HLA-A, B, C, DPB1, DQB1 and DRB1 alleles were assigned according to the HLA sequencing based typing methodology as previously described^28^. In brief, the amplicons containing HLA fragments were purified using ExoSAP-IT (Atria Genetics) and sequenced in both directions. The HLA alleles were assigned at a 4-digit level using the ASSIGN 3.5 software (Conexio Genomics, Perth, WA, Australia). Any ambiguous results were applied to additional exon sequencing^48^. The HLA-A, B and DRB1 genotype for some of the persistent infection donors were extracted from our previous report^28^, while the others were first reported in this study.

### Statistical analysis

The frequency of *IL28B* SNP and HLA alleles was calculated by direct counting from the sequencing results. Possible age difference between the HCV spontaneous clearance group versus the persistent infection group was examined by the T-test. Individual association of gender, ethnicity, *IL28B* SNP and HLA alleles with HCV clearance was examined by the Chi-square test or Fisher’s exact test when Chi-square test can not applied. Association strength was calculated by OR with 95% CI and statistical *P*-value. The Bonferroni correction was applied for multiple comparisons. Statistically significant association was indicated when the *P*-value was less than 0.05. Multivariate logistic regression using forward conditional stepwise method was applied to identify independent factors associated with HCV clearance. Statistical analyses were performed in SPSS version 16.0 (SPSS Inc, Chicago, IL, USA).

## Additional Information

**How to cite this article**: Huang, J. *et al*. The Associations of HLA-A*02:01 and DRB1*11:01 with Hepatitis C Virus Spontaneous Clearance Are Independent of *IL28B* in the Chinese Population. *Sci. Rep.* **6**, 31485; doi: 10.1038/srep31485 (2016).

## Supplementary Material

Supplementary Information

## Figures and Tables

**Table 1:** Characteristics of the blood donors with cleared and persistent HCV infection.

Characteristic	Donors with HCV infection		*P*
	Spontaneous clearance (n = 231)	Persistent infection (n = 429)	
Age Median (Range)	24 (18–53)	31 (18–55)	3.29E-4
Gender			
Male n(%)	152 (65.8)	355 (82.8)	8.58E-7
Female n(%)	79 (34.2)	74 (17.2)	
Ethnicity			
Chinese Han n(%)	227 (98.3)	428 (99.8)	0.053
Chinese others n(%)	4 (1.7)	1 (0.2)	

**Table 2:** Allelic distribution of HLA class I alleles and their correlation with HCV infection outcome in Chinese population.

HLA allele	Spontaneous clearance n (%)	Persistent infection n (%)	*P* (*Pc*)	OR (95%C.I.)
A*02:01	**47 (10.2)**	**52 (6.1)**	**0.004 (0.024)**	**1.839 (1.212, 2.791)**
A*02:03	33 (7.1)	65 (7.6)	0.888	0.969 (0.624, 1.504)
A*02:07	54 (11.7)	101 (11.8)	0.883	1.027 (0.718, 1.470)
A*11:01	122 (26.4)	251 (29.3)	0.414	0.895(0.684, 1.169)
A*24:02	53 (11.5)	131 (15.3)	0.083	0.735 (0.519, 1.042)
A*33:03	51 (11.0)	89 (10.4)	0.572	1.113 (0.768, 1.612)
B*13:01	38 (8.2)	67 (7.8)	0.581	1.129 (0.734, 1.737)
B*15:02	39 (8.4)	64 (7.5)	0.351	1.227 (0.797, 1.889)
B*40:01	55 (11.9)	137 (16.0)	0.077	0.724 (0.506, 1.036)
B*46:01	72 (15.6)	134 (15.6)	0.689	1.072 (0.764, 1.504)
B*58:01	44 (9.5)	83 (9.7)	0.832	1.045 (0.699, 1.562)
C*01:02	87 (18.9)	173 (20.3)	0.783	0.959 (0.710, 1.295)
C*03:02	43 (9.3)	80 (9.4)	0.84	1.042 (0.700, 1.549)
C*03:04	59 (12.8)	110 (12.9)	0.819	1.041 (0.735, 1.475)
C*06:02	29 (6.3)	42 (4.9)	0.217	1.363 (0.832, 2.231)
C*07:02	61 (13.3)	151 (17.7)	0.062	0.729 (0.523, 1.017)
C*08:01	54 (11.7)	86 (10.1)	0.234	1.251 (0.865, 1.811)

Alleles with >5% frequencies are shown at the 4-digit level and analyzed; *Pc*, Corrected *P* value for multiple comparisons by Bonferroni correction. Alleles with statistical significance after Bonferroni correction are indicated in bold.

**Table 3:** Allelic distribution of HLA class II alleles and their correlation with HCV infection outcome in Chinese population.

HLA allele	Spontaneous clearance n (%)	Persistent infection n (%)	*P* (*Pc*)	OR (95%C.I.)
DPB1*02:01	73 (16.1)	145 (17.0)	0.914	0.983 (0.716, 1.349)
DPB1*02:02	24 (5.3)	52 (6.1)	0.671	0.897 (0.543, 1.482)
DPB1*04:01	39 (8.6)	70 (8.2)	0.643	1.103 (0.728, 1.671)
DPB1*05:01	178 (39.2)	340 (39.8)	0.695	1.053 (0.812, 1.367)
DPB1*13:01	29 (6.4)	65 (7.6)	0.527	0.862 (0.545, 1.364)
DQB1*02:01	31 (6.7)	60 (7.0)	0.651	0.901 (0.573, 1.417)
DQB1*02:02	33 (7.2)	38 (4.4)	0.063	1.576 (0.972, 2.557)
DQB1*03:01	121 (26.3)	167 (19.5)	0.017 (0.136)	1.398 (1.060, 1.843)
DQB1*03:02	27 (5.9)	46 (5.4)	0.892	1.035 (0.633, 1.693)
DQB1*03:03	77 (16.7)	126 (14.7)	0.579	1.093 (0.798, 1.499)
DQB1*05:02	**38 (8.3)**	**113 (13.2)**	**0.002 (0.016)**	**0.547 (0.370, 0.809)**
DQB1*06:01	41 (8.9)	84 (9.8)	0.394	0.842 (0.567, 1.251)
DQB1*06:02	22 (4.8)	52 (6.1)	0.227	0.729 (0.436, 1.220)
DRB1*03:01	31 (6.7)	60 (7.0)	0.626	0.893 (0.565, 1.410)
DRB1*04:05	16 (3.5)	52 (6.1)	0.022 (0.176)	0.514 (0.288, 0.916)
DRB1*07:01	35 (7.6)	47 (5.5)	0.214	1.338 (0.844, 2.120)
DRB1*08:03	23 (5.0)	44 (5.1)	0.715	0.907 (0.537, 1.531)
DRB1*09:01	77 (16.7)	117 (13.6)	0.281	1.197 (0.863, 1.659)
DRB1*11:01	44 (9.5)	45 (5.2)	0.007 (0.056)	1.820 (1.172, 2.826)
DRB1*12:02	56 (12.1)	96 (11.2)	0.9	1.024 (0.713, 1.470)
DRB1*15:01	43 (9.3)	107 (12.5)	0.031 (0.248)	0.657 (0.448, 0.964)

Alleles with >5% frequencies are shown at the 4-digit level and analyzed; *Pc*, Corrected *P* value for multiple comparisons by Bonferroni correction. Alleles with statistical significance after Bonferroni correction are indicated in bold.

**Table 4:** Association of *IL28B* (rs8099917) with HCV infection outcome in Chinese population.

rs8099917	Spontaneous clearance n (%)	Persistent infection n (%)	*P*	OR (95%C.I.)
Allele				
T	451 (97.6)	783 (91.3)	7.97E-6	3.927 (2.064, 7.473)
G	11 (2.4)	75 (8.7)		
Genotype				
TT	221 (95.7)	354 (82.5)	1.50E-6 (TT vs. TG + GG)	4.682 (2.370, 9.250)
TG	9 (3.9)	75 (17.5)		
GG	1 (0.4)	0 (0)		

**Table 5:** Logistic regression analysis of variables associated with HCV spontaneous clearance.

Variables	OR (95% CI)	*p* value
Age	1.035 (1.021, 1.048)	3.02E-7
Gender (M/F)	0.405 (0.310, 0.531)	5.05E-11
Rs8099917 (T/G)	3.842 (1.993, 7.405)	5.82E-5
HLA-A*02:01	1.798 (1.169, 2.766)	0.008
HLA-DQB1*03:01	Not enter the equation	0.244
HLA-DQB1*05:02	Not enter the equation	0.051
HLA-DRB1*04:05	Not enter the equation	0.069
HLA-DRB1*11:01	1.921 (1.223, 3.017)	0.005
HLA-DRB1*15:01	Not enter the equation	0.305

**Table 6:** Summary of HLA alleles with HCV clearance or persistence in published reports and this study.

Associated HLA allele	Association with HCV	population (clearance or SVR/persistence or NR, if not specified)	Study
A*02	Persistence in Caucasian; spontaneous clearance in non-Caucasians	USA (49/56)	27
	SVR	Chinese (144 A*02 positive/149 A*02 negative patients)	33
	Protective	USA (11,728 HCV uninfected/5901 HCV infected)	34
A*02:01	Spontaneous clearance*	Chinese (231/429)	This study
A*02:03	No association	Chinese (231/429)	This study
A*02:07	No association	Chinese (231/429)	This study
B*57	Spontaneous clearance	USA (231/444)	23
	Spontaneous clearance	USA(136/622)	25
	Spontaneous clearance	USA (66/280)	29
	Spontaneous clearance	West Africa (35/37)	30
	No association	USA (79/200)	35
	No association	Chinese (231/429)	This study
DQB1*03	Spontaneous clearance	USA (49/56)	27
	Protective	USA (11,728 HCV uninfected/5901 HCV infected)	34
	Protective	Japan (2,963 healthy controls/481 chronic HCV infected)	42
DQB1*03:01	Spontaneous clearance	USA (200/374)	26
	Spontaneous clearance	Italy (49/68)	41
	Spontaneous clearance	Iran (54/63)	38
	Spontaneous clearance	Chinese (432/686)	43
	SVR	Chinese (156/87)	43
	SVR	Pakistan (150/54)	36
	Clearance	Meta-analysis	37
	Clearance	Meta-analysis	7
DQB1*03:01	No association	Chinese (231/429)	This study
DQB1*05	Spontaneous clearance	USA (49/56)	27
DQB1*05:01	Spontaneous clearance	USA (200/374)	26
DQB1*05:02	Clearance	Italy (30 exposed but uninfected/116 HCV infected)	45
DQB1*05:02	No association	Chinese (231/429)	This study
DRB1*11	Clearance	Meta-analysis	7
	Spontaneous clearance	Iran (54/63)	38
	SVR	Pakistan (150/54)	36
	Protective	USA (11,728 HCV uninfected/5901 HCV infected)	34
	Protective	Italy (70 HCV uninfected/73 HCV seropositive)	39
DRB1*11:01	Spontaneous clearance	UK (85/170)	40
	Clearance	Meta-analysis	37
DRB1*11:01	Spontaneous clearance	Chinese (231/429)	This study
DRB1*15	Spontaneous clearance	Ireland (86/141)	24
	Spontaneous clearance	Germany (21/49)	46
	Persistence	Tunisia (24/75)	47
DRB1*15:01	No association	Chinese (231/429)	This study

^*^The significance of the association in this study was determined by the results of logistic regression analysis.
