# Supplementary material for: The Associations of HLA-A*02:01 and DRB1*11:01 with Hepatitis C Virus Spontaneous Clearance Are Independent of IL28B in the Chinese Population
Source: Sci Rep. 2016 Aug 11;6:31485. doi: 10.1038/srep31485 (PMC4980596; doi:10.1038/srep31485)
Supplement: Supplementary Information [file srep31485-s1.pdf]

The Associations of HLA-A*02:01 and DRB1*11:01 with Hepatitis C Virus Spontaneous Clearance Are Independent of *IL28B* in the Chinese Population

Jieting Huang[1,2*], Ke Huang[1,2*], Ru Xu[1,2*], Min Wang[1,2], Qiao Liao[1,2], Huaping Xiong[1,2], Chengyao Li[3], Xi Tang[3], Zhengang Shan[1,2], Ming Zhang[4], Xia Rong[1,2], Kenrad Nelson[5], Yongshui Fu[1,2]

Table S1. Clearance rate of the study population

Gender	Spontaneous clearance	Persistent infection	Clearance rate (%)	*P* (male/female)	OR (95%C.I.)
Male	152	1046	12.7	2.03E-9	0.397 (0.292, 0.541)
Female	79	216	26.8		
Total	231	1262	15.5		

Table S2. Subjects with available HLA genotyping results

HLA	Spontaneous clearance	Persistent infection
A	231	429
B	231	429
C	230	427
DPB1	227	427
DQB1	230	429
DRB1	231	429

Table S3. Association of *IL28B* rs12979860 with HCV spontaneous clearance in Chinese population

rs12979860	Spontaneous clearance n (%)	Persistent infection n (%)	*P*	OR (95%C.I.)
Allele				
C	451 (97.6)	784 (91.4)	1.04E-5	3.870 (2.033, 7.368)
T	11 (2.4)	74 (8.6)		
rs12979860			CC vs CT+TT	
CC	221 (95.7)	355 (82.8)		
CT	9 (3.9)	74 (17.2)	2.03E-6	4.607 (2.331, 9.106)
TT	1 (0.4)	0 (0)		

Table S4. The associations of HLA alleles with HCV spontaneous clearance stratified by their *IL28B* genotype

HLA	*IL28B* TT (n=575)				*IL28B* TG+GG (n=85)			
	Spontaneous clearance (2n=442)	Persistent infection (2n=708)	*P*	OR (95% CI)	Spontaneous clearance (2n=20)	Persistent infection (2n=150)	*P*	OR (95% CI)
A*02:01	47 (10.6)	47 (6.6)	0.016	1.673 (1.096, 2.555)	0 (0)	5 (3.3)	0.999	-
DQB1*03:01	118 (26.8)	135 (19.1)	0.002	1.555 (1.173, 2.063)	3 (15.0)	32 (21.3)	0.769	0.651 (0.179, 2.359)
DQB1*05:02	36 (8.2)	94 (13.3)	0.008	0.582 (0.389, 0.872)	2 (10.0)	19 (12.7)	0.999	0.766 (0.165, 3.567)
DRB1*04:05	16 (3.6)	48 (6.8)	0.023	0.516 (0.290, 0.921)	0 (0)	4 (2.7)	0.999	-
DRB1*11:01	42 (9.5)	38 (5.4)	0.007	1.851 (1.173, 2.921)	2 (10.0)	7 (4.7)	0.286	2.270 (0.438, 11.774)
DRB1*15:01	41 (9.3)	89 (12.6)	0.086	0.711 (0.481, 1.051)	2 (10.0)	18 (12.0)	0.999	0.815 (0.174, 3.807)

Table S5. Prediction of HCV spontaneous clearance

Genotype	Sensitivity	Specificity	Positive predictive value	Negative predictive value
IL28B	22.9	90.7	57.0	68.6
HLAs	26.0	95.1	55.8	70.5
IL28B+HLAs	28.1	88.9	57.8	69.7

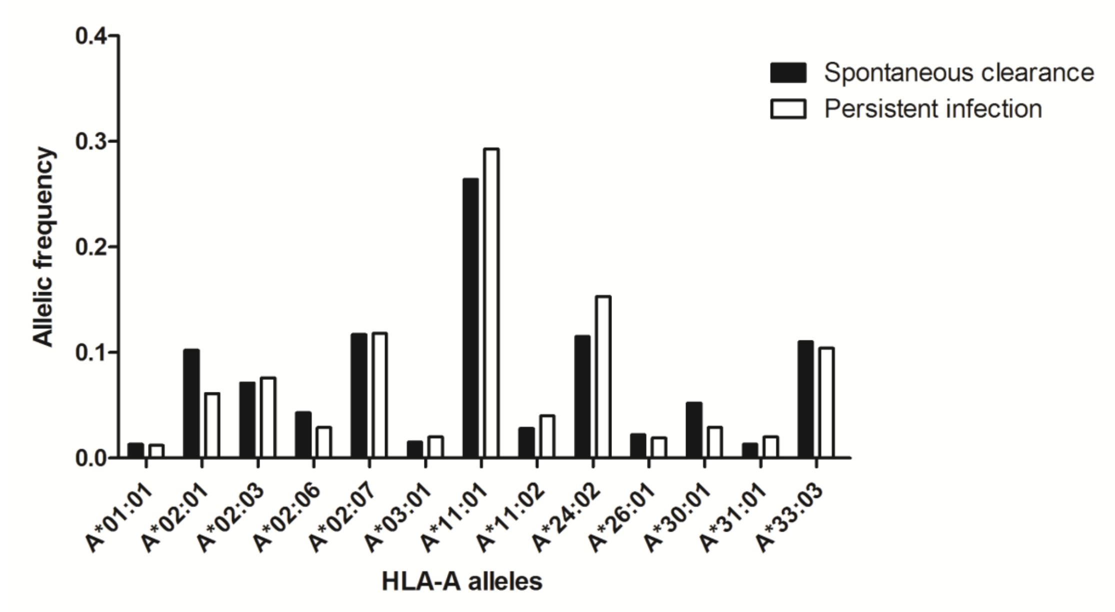

Figure S1. HLA-A diversity between the subjects with HCV clearance and persistent infection. Alleles with frequencies higher than 1.0% are shown.

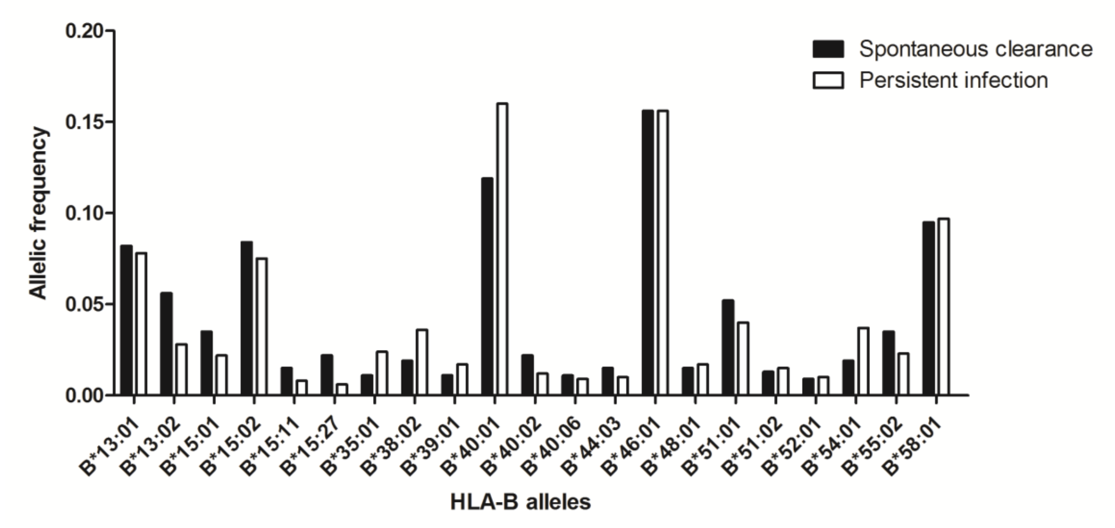

Figure S2. HLA-B diversity between the subjects with HCV clearance and persistent infection. Alleles with frequencies higher than 1.0% are shown.

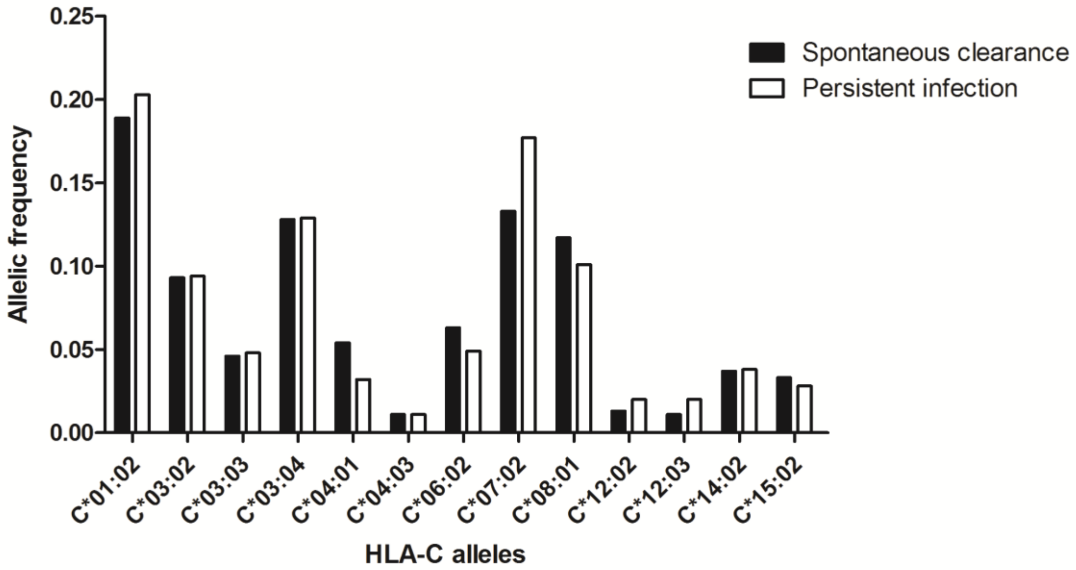

Figure S3. HLA-C diversity between the subjects with HCV clearance and persistent infection. Alleles with frequencies higher than 1.0% are shown.

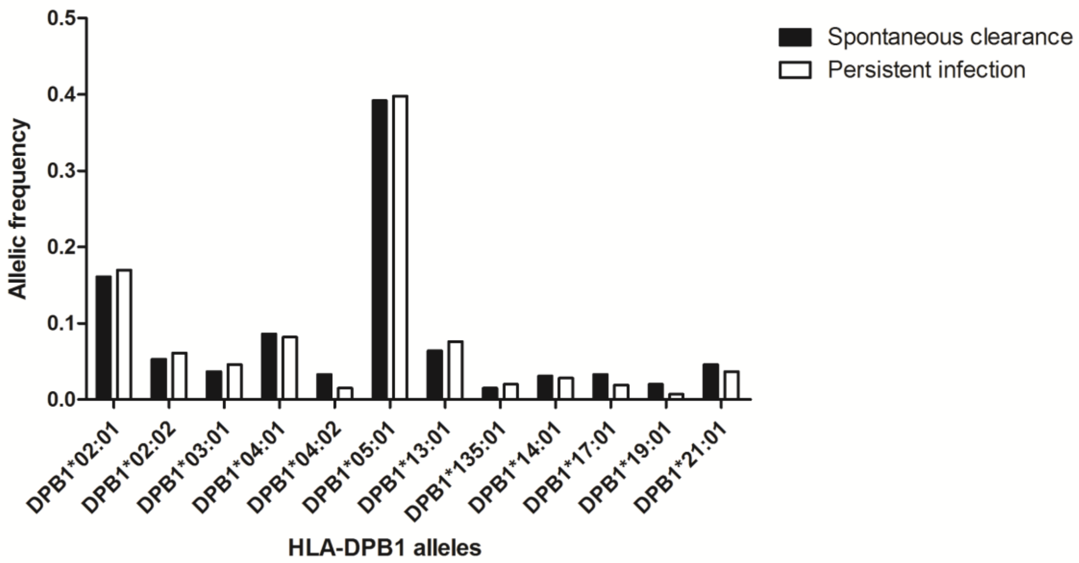

Figure S4. HLA-DPB1 diversity between the subjects with HCV clearance and persistent infection. Alleles with frequencies higher than 1.0% are shown.

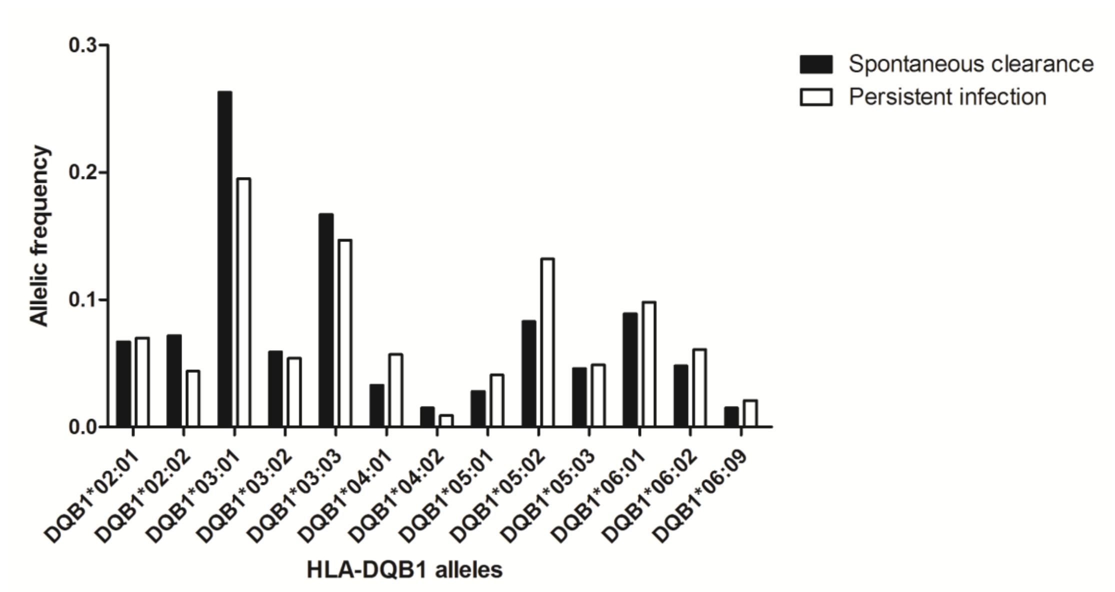

Figure S5. HLA-DQB1 diversity between the subjects with HCV clearance and persistent infection. Alleles with frequencies higher than 1.0% are shown.

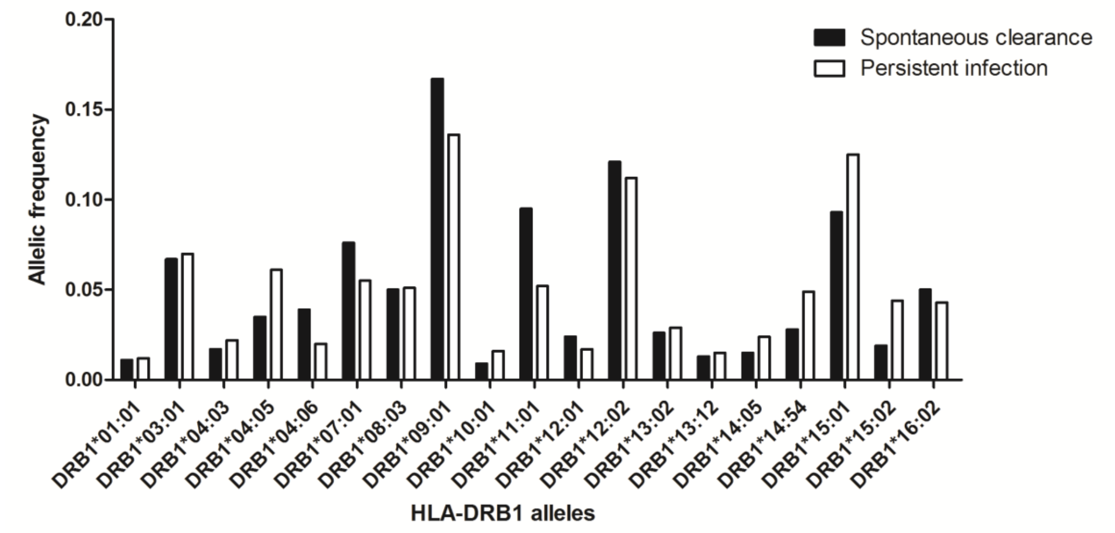

Figure S6. HLA-DRB1 diversity between the subjects with HCV clearance and persistent infection. Alleles with frequencies higher than 1.0% are shown.
